# Insights into Arbutin Effects on Bone Cells: Towards the Development of Antioxidant Titanium Implants

**DOI:** 10.3390/antiox9070579

**Published:** 2020-07-02

**Authors:** Maria A. Bonifacio, Giorgia Cerqueni, Stefania Cometa, Caterina Licini, Luigia Sabbatini, Monica Mattioli-Belmonte, Elvira De Giglio

**Affiliations:** 1 Department of Chemistry, University of Bari Aldo Moro, 70126 Bari, Italy; maria.bonifacio@uniba.it (M.A.B.); luigia.sabbatini@uniba.it (L.S.); 2 Jaber Innovation s.r.l., 00144 Roma, Italy; stefania.cometa@jaber.it; 3 DISCLIMO Department of Clinical and Molecular Sciences, Università Politecnica delle Marche, 60126 Ancona, Italy; g.cerqueni@pm.univpm.it (G.C.); caterina.licini@polito.it (C.L.); m.mattioli@staff.univpm.it (M.M.-B.); 4 DISAT—Department of Applied Science and Technology, Polytechnic of Turin, 10129 Turin, Italy

**Keywords:** arbutin, oxidative stress, coating, surface characterization, titanium implant, bone cells, MSCs, viability, differentiation

## Abstract

Arbutin is a plant-derived glycosylated hydroquinone with antioxidant features, exploited to combat cell damage induced by oxidative stress. The latter hinders the osseointegration of bone prostheses, leading to implant failure. Little is known about arbutin antioxidant effects on human osteoblasts, therefore, this study explores the in vitro protective role of arbutin on osteoblast-like cells (Saos-2) and periosteum-derived progenitor cells (PDPCs). Interestingly, cells exposed to oxidative stress were protected by arbutin, which preserved cell viability and differentiation. Starting from these encouraging results, an antioxidant coating loaded with arbutin was electrosynthesized on titanium. Therefore, for the first time, a polyacrylate-based system was designed to release the effective concentration of arbutin in situ. The innovative coating was characterized from the physico-chemical and morphological point of view to achieve an optimized system, which was in vitro tested with cells. Morpho-functional evaluations highlighted the high viability and good compatibility of the arbutin-loaded coating, which also promoted the expression of PDPC differentiation markers, even under oxidative stress. These results agreed with the coatings’ in vitro antioxidant activity, which showed a powerful scavenging effect against DPPH radicals. Taken together, the obtained results open intriguing opportunities for the further development of natural bioactive coatings for orthopedic titanium implants.

## 1. Introduction

Oxidative stress is correlated with aging and with several age-related bone pathological conditions. It alters bone remodeling, inducing the apoptosis of osteoblasts and osteocytes. This process ultimately leads to several bone diseases, first and foremost, osteoporosis [1]. In addition, increased oxidative stress occurs after the implantation of metallic prostheses, which represents the most common treatment to guarantee suitable load bearing features. Thus, to prompt implant osteointegration, it is useful to protect bone cells from oxidative stress, which worsens inflammation and could cause implant rejection [2]. Cells innately counteract the adverse effects of reactive oxygen species (ROS) by several mechanisms, including the upregulation of free radical scavenger enzymes, via the activation of a family of ubiquitous transcription factors known as Forkhead box O (FoxO) [3]. To help cells restore the oxidative balance, one of the most innovative research strategies focuses on the exploitation of plant-derived antioxidant molecules, to alleviate the negative effects of free radicals [4]. Arbutin is a glycosylated hydroquinone typical of the Ericaceae family (i.e., blueberries, bearberries, cranberries, uva ursi), but it is also found in wheat, strawberries and pear trees. This phytochemical, widely known for its skin-lightening properties [5], has been also used as an antimicrobial compound [6]. More recently, a few researchers studied arbutin antioxidant features, exploring the potential usage of this molecule in different medical applications. Dadgar and coworkers assessed the arbutin reduction of oxidative stress in an in vivo model of Parkinson’s disease [7], while Zao et al. described an arbutin-mediated attenuation of in vitro oxidative injury, induced by H_2_O_2_ on retinal ganglion cells [8]. As far as bone diseases are concerned, arbutin inhibited in vitro osteoclasts’ differentiation, suppressing RANK-L-mediated superoxide production, which is one of the main sources of ROS in osteoporotic tissues [9,10]. Furthermore, a positive effect of arbutin was demonstrated on mouse osteoblasts’ in vitro proliferation and differentiation [11]. However, to the best of our knowledge, arbutin antioxidant effects on human osteoblasts and their precursors are still unexplored. Therefore, this work aims at unveiling arbutin scavenger activity on human osteoblast-like cells exposed to oxidative stress. Firstly, the most effective arbutin concentration eliciting antioxidant activity was established with an in vitro screening on Saos-2 and periosteal-derived precursor cells (PDPCs). In a second step, the selected arbutin amount was loaded into a polymeric coating, synthetized on titanium implants. Thus, delivering arbutin in situ would target bone cells, preventing high oral dosage administration and rapid clearance [12]. To date, no arbutin-loaded coatings on metallic implants are documented in the literature. Nevertheless, Córdoba and co-workers envisioned the opportunity to graft flavonoid moieties on titanium surfaces to reduce free radical damage [13]. However, a covalent grafting would hinder the release of bioactive molecules from the coating, limiting their benefits. On the other hand, Chen et al. investigated the opportunity to coat titanium implants with catechol, the ortho isomer of hydroquinone, to suppress ROS toxicity [14]. The authors developed a multilayered system, depositing a film of chitosan–catechol conjugate on titanium through spin-coating. However, this technique could not allow for finely tuning the coating thickness and homogeneity. Therefore, in the present work, an electrochemical polymerization was chosen to obtain a stable, uniform and adherent coating on titanium, able to release arbutin once implanted. A polyacrylate-based copolymer [15] was selected to provide an anticorrosion, cytocompatible coating, which was loaded with arbutin during or after its electrochemical growth on titanium. The developed coating was characterized by X-ray photoelectron spectroscopy (XPS), scanning electron microscopy (SEM) and high-performance liquid chromatography (HPLC) analyses, while its in vitro antioxidant activity was assessed by a 2,2-difenil-1-picrylhydrazyl (DPPH) assay, a free radical scavenging method assay. The prepared coating, beyond being cytocompatible, reduced the oxidative stress in vitro, helping to prevent post-implant complications and accelerating implant osseointegration.

## 2. Materials and Methods

### 2.1. Materials

All chemicals were supplied by Sigma-Aldrich^®^ (Italy) unless otherwise specified. They were used without further purification. The poly (ethylene-glycol diacrylate), PEGDA (M_n_ 575 Da), was used as macromer, while acrylic acid (AA) was the monomer, to obtain PEGDA-AA copolymer coatings. Ammonium peroxydisulfate ((NH_4_)_2_S_2_O_8_) was selected as an electrochemical initiator. The purity of arbutin (hydroquinone β-d-glucopyranoside) was greater than 99%.

### 2.2. Coating Preparation

Titanium sheets (2 cm^2^) were used as electrodes, after mechanical polishing by fine diamond paper, Al_2_O_3_ powder (50 µm) and ultrasonical etching in ethanol and, successively, triple-distilled water. The PEGDA macromer 0.1 M was co-polymerized with AA monomer 0.05 M, in the presence of the electrochemical initiator (NH_4_)_2_S_2_O_8_. Cyclic voltammetry was set up to obtain a PEGDA-AA coating, as reported in detail in [16]. Briefly, for all the electrochemical deposition steps, a three-electrode cell was assembled and connected to a PAR VersaSTAT4 potentiostat–galvanostat (Princeton Applied Research, UK). A platinum wire was used as an anode, while the cathode was the titanium electrode. An Ag/AgCl (KCl sat.) in water (0.199V vs. SHE at 25 °C) was the reference system. Moreover, in order to improve the compactness of the PEGDA-AA coatings, after arbutin loading (see Section 2.3 for details), they were subjected to an annealing treatment, as already reported [17]. Briefly, the annealing was carried out in air at 200 °C for 10 min. In order to ascertain the thermal stability of the biomolecule during the heat treatment, a thermogravimetric analysis of arbutin was carried out by heating 5–10 mg of sample in an air-saturated atmosphere, using a PerkinElmer TGA-400 instrument (PerkinElmer Inc., Waltham, MA). The heat range was set between 30 and 600 °C at a flow rate of 20 °C/min. The gas flow was set at 20mL/min. Thermograms (TG) with respective derivative curves (DTG) were recorded and data were analyzed using the software TGA Pyris series. Additional information is reported in the Supplementary Materials.

### 2.3. Arbutin Loading Procedures

Arbutin was embedded within coatings using two different procedures: during electrosynthesis (DE) and after electrosynthesis (AE), exploiting the same approach described in [16]. In the first case, the drug was trapped in the copolymer coating during its electrosynthesis from an electrolyte solution containing the natural compound (ranging from 0.1 M to 0.01 M), together with the monomer/macromer and the electrochemical initiator. In the AE loading procedure, the coatings were first electropolymerized on titanium sheets and successively dipped, for 1 h, in an aqueous solution with arbutin in the range 0.1–0.01 M. Finally, the sheets were removed from the solution and dried with a nitrogen flux. The fine-tuning of both the arbutin concentration and loading time allowed the achievement of the optimum balance between cytocompatibility and antioxidant features.

### 2.4. X-ray Photoelectron Spectroscopy (XPS)

A scanning microprobe PHI 5000 VersaProbe II, equipped with a monochromatized AlKα X-ray radiation source (Physical Electronics, Chanhassen, MN), was exploited to perform XPS analysis. The samples were analyzed in HP mode (scanned size ~1400 × 200 µm), with an X-ray take-off angle of 45°. For each sample, survey scans and high-resolution spectra were recorded in FAT mode (pass energy 117.4 eV and 29.35 eV, respectively). The instrument base pressure was 10^−9^ mbar.

The MultiPak software package (version 9.9.0), a non-linear least square fitting program, was exploited to fit the detailed spectra through Gaussian–Lorentzian peaks with the same full width at half maximum (FWHM). The lower binding energy of C1s photo peak (e.g., C1s hydrocarbon peak) was set at 284.8 eV as a charge reference. Normalized peak areas were used to quantify atomic percentages (At%). Empirically derived sensitivity factors, in accordance with the MultiPak library, enabled the corrections to compare data from different elements and to normalize the peak areas.

### 2.5. Scanning Electron Microscopy (SEM) Morphological Analysis of the Coatings

SEM analysis was performed on unannealed and annealed PEGDA-AA coatings loaded with arbutin after electrosynthesis, using a Philips XL 20 scanning electron microscope (FEI Italia S.r.l., Milan, Italy). The specimens were mounted on aluminum stubs and were gold-sputtered before the analysis.

### 2.6. High-Performance Liquid Chromatography (HPLC)

Arbutin loading and release from the coatings were monitored by HPLC (Prominence Series 20 with SPD-M20A PDA detector, Shimadzu) following the method reported by Muchtaridi et al., with some modifications [18]. A Shim-Pack GIST C18-AQ column (150 mm × 4.6 mm, 5 μm Shimadzu) was eluted in isocratic mode at 30 °C, with 15% methanol in water, monitoring the effluent at 282 nm. The mobile phase’s flow rate was kept at 1 mL/min and the sample was injected through a 20 µL injection loop. Using LabSolutions software, a calibration curve was also built to quantify the loading and release of arbutin from the prepared coatings. The release experiment was performed after incubating titanium-coated samples in 0.9% NaCl solution for 10 min, 30 min, 1 h, 2 h, 4 h, 8 h and 24 h.

### 2.7. DPPH Assay

According to the procedure already described by Kudachikar et al., arbutin’s in vitro antioxidant activity was assessed by DPPH assay [19]. A stock solution of DPPH 60 μM was prepared in methanol and its absorbance was measured at 515 nm using a Cary 60 UV–Vis Spectrophotometer (Agilent Technologies, Santa Clara, CA, USA). A calibration curve with arbutin standard solutions ranging from 0.4 mM to 8 µM (R^2^ = 0.999) was built and exploited for quantifications. Arbutin-containing coatings (PEGDA-AA/Arb AE 1h with and without annealing, PEGDA-AA/Arb DE) were incubated with DPPH at room temperature, in dark conditions and absorbance changes were measured after 30 min. Incubation time was selected after an optimization procedure achieved through the continuous monitoring of absorbance decay at 515 nm. The radical scavenging activity percentage (%RSA) was calculated with the following equation:

%RSA = (ADPPH − AS)/ADPPH 100(1)

in which A_S_ represents the sample’s absorbance, while A_DPPH_ is the absorbance of bare DPPH [20]. Each measurement was performed in triplicate.

### 2.8. Cell Culture, Cytocompatibility and Antioxidant Activity Assessment

#### 2.8.1. Cell Cultures

Saos-2 cell line (ATCC-HTB-85) was cultured in high/low glucose (1:1) Dulbecco’s modified Eagle’s medium (DMEM) (H-DMEM; Sigma-Aldrich, St. Louis, MO, USA; L-DMEM; Euroclone, Milan, Italy) supplemented with 10% fetal bovine serum (FBS; Corning Inc., Corning, NY, USA ) and 100 U/mL penicillin–streptomycin (Thermo Fisher Scientific, Waltham, MA, USA) at 37 °C in a humidified atmosphere, with 5% CO_2_. The periosteal derived precursor cells (PDPCs) were harvested from the periosteal tissue of healthy subjects undergoing surgery for orthopedic trauma, as previously described [21]. According to the Local Ethical Committee guidelines and the 1964 Helsinki declaration, informed consent was obtained. Patients were aware of the voluntariness of their participation in the study and that the tissue used for the research was a discard of surgical procedures. Briefly, periosteal explants were washed thrice with phosphate-buffered saline (D-PBS) lacking in Ca^2+^ and Mg^2+^, aseptically cut into small pieces (4–9 mm^2^) and then positioned in a 100 mm culture dish in DMEM/F-12 supplemented with 10% FBS and 1% penicillin–streptomycin (100 U/mL) in a humidified incubator at 37 °C with 5% CO_2_. The medium was changed every 2–3 days and cells were characterized for their mesenchymal stem/stromal cell (MSC) origin, according to the minimal criteria of the International Society for Cellular Therapy (ISCT) [22].

#### 2.8.2. Arbutin Treatment

Saos-2 and PDPCs from the 3rd passage of subculture were seeded into 96/well plates at a concentration of 1 × 10^4^ cells/cm^2^. After 24 h, each appropriate medium was changed with the one containing 0.1, 0.2 or 0.4 mM of arbutin and cultured at 37 °C with 5% CO_2_ for up to 72 h. Increasing concentrations of H_2_O_2_ (from 25 µM to 300 µM) were tested on both cell populations to induce oxidative stress. In order to assess the potential of arbutin in preventing oxidative stress, H_2_O_2_ was added after 24 h to the cell cultures containing arbutin and cell viability was assessed 24 h (T1) and 48 h (T2) after stress induction. Control cultures (Ctrl) were represented by Saos-2 and PDPCs cultured in their appropriate media without arbutin. To evaluate the arbutin effect on PDPC gene and protein expressions, arbutin was maintained up to 14 days in the presence of normal or differentiating medium. Normal medium, defined as complete medium (CM), was represented by DMEM/F-12 supplemented with 10% FBS and 1% penicillin–streptomycin (100 U/mL). Differentiating medium (DM) consisted of 0.1 mM dexamethasone, 10 mM β-glycerophosphate and 0.05 mM ascorbic acid in DMEM/F-12 supplemented with 10% FBS and 1% penicillin–streptomycin (100 U/mL), as previously described [23].

#### 2.8.3. Material Seeding

The coated Ti specimens were UV-sterilized (254 nm) for 48 h (24 h per side). PDPCs were detached using 0.25% trypsin in 1 mM EDTA and plated in triplicate onto: (i) PEGDA-AA Ann. (internal controls) or (ii) PEGDA-AA/Arb AE Ann. at a density of 1 × 10^4^ cells/cm^2^.

#### 2.8.4. MTT (3-Dimethylthiazol-2,5-diphenyltetrazolium bromide) Colorimetric Assay

An MTT viability assay was performed after culturing Saos-2 and PDPCs in the presence of different arbutin concentrations, as well as after cell seeding on the different Ti substrates. Briefly, the medium was removed, 200 μL of MTT (Aldrich 135038) solution (5 mg/mL in DMEM without phenol red) and 1.8 mL DMEM were added to all cell monolayers. Then, the multi-well plates were incubated at 37 °C for 4 h. After discarding the supernatants, the dark blue formazan crystals were dissolved by adding 2 mL of solvent (4% HCl 1N in isopropanol absolute) and quantified by spectrophotometry (MultiskanGo, Thermo Scientific™), monitoring the absorbance at 570 and 690 nm.

#### 2.8.5. qRT-PCR

Total RNA was retrieved from PDPCs cultured for 7 and 14 days, with or without arbutin, using the PerfectPure RNA cultured cell kit (5-Prime GmbH, Hamburg, Germany) according to the manufacturer’s instructions. UV spectrophotometric analysis (bioPhotometer plus, Eppendorf GmbH, Germany) was used for the evaluation of RNA quality and quantitation. An amount equal to 2.5 µg of total RNA was reverse transcribed in a 20 µL reaction volume using the SuperScript IV VILO Master Mix (Thermo Fisher, Monza, Italy). Neo-synthesized cDNA was kept at −20 °C. Real-time assays with SsoFast™ EvaGreen^®^ Supermix (1× in a final volume of 10 µL) were performed in a Mastercycler Realplex2 thermocycler (Eppendorf GmbH, Germany). All PCR reactions included 1 µL of cDNA (equivalent to 50 ng of total RNA template). Primer sequences were designed by Primer 3 (v. 4.1.0) software and each primer was used at a 200 nM final concentration. To circumvent any substantial homology to pseudo-genes or other unexpected targets, primer specificity was tested by BLAST against RefSeq Genomes. The mRNA of both reference genes (gusb and gapdh) and each gene of interest (bmp2, runx-2, coll1a1, alp and sparc) were measured under matching conditions and at the same time in each assay. Supplementary Materials Table S1 depicts oligonucleotide sequences designed for the target and reference genes. Primers exhibited equal amplification efficiency. The cycling conditions included an initial step at 95 °C for 30 s, followed by 40 cycles at 95 °C for 5 s and 60 °C for 20 s. The specificity of the PCR reactions was also determined by melt curve analysis. Indeed, for each amplicon, the detected melting temperature was the expected one. Threshold cycle (Ct) values for reference genes were utilized to normalize cell mRNA data. Each assay was made in triplicate. Normalization involved the ratio of mRNA concentrations for specific genes of interest (as mentioned above) to that corresponding to Ct medium values for glyceraldehyde-3-phosphate dehydrogenase (GAPDH) and beta-glucuronidase (GUSB) [24]. Data were expressed as gene relative expression (2^−ΔCt^). The qPCR efficiency in all experiments was more than 90%. The difference between the actual and theoretical (100%) efficiencies would result in an underestimation of the mRNA concentration of all the analyzed samples. To point out the effect of arbutin on PDPC differentiation ability, the ΔΔCt method for fold change evaluation was used [25], comparing values obtained in cells cultured with arbutin with those cultured without arbutin and data obtained with or without oxidative stress.

#### 2.8.6. Western Blot Analysis

Total proteins were extracted from PDPCs after 7 and 14 days of culture with or without arbutin using the RIPA Lysis Buffer System (PBS, 1% Nonidet P-40, 0.5% sodium deoxycholate, 0.1% SDS, 0.004% sodium azide) supplemented with protease inhibitors (S8820, Sigma-Aldrich, St. Louis, MO, USA). In cells cultured on Ti, total proteins were extracted using TRIzol reagent (Invitrogen, Carlsbad, CA, USA) according to the manufacturer’s instructions. Protein concentration was measured by Bradford reagent (B6916, Sigma-Aldrich). Total protein extracts (30 µg) were incubated with NuPAGE™ LDS Sample Buffer (4X) (Invitrogen) according to the manufacturer’s instructions, fractionated in NuPAGE™ 4–12% Bis-Tris Protein Gels (Invitrogen) and electrophoretically transferred to PVDF membranes (Millipore). Membranes were incubated with 5% milk in Tris-buffered saline with 0.1% Tween 20 (TBS-T) to block non-specific sites and then with rabbit anti-BMP2, anti-ALP (57, 78 and 200 kDa fragments), anti-type I collagen, mouse anti-RUNX-2 and anti-osteonectin (ON, 35 and 45 kDa fragments) primary antibodies at 4 °C. Mouse anti-GAPDH was used as an endogenous control. After overnight incubation, the membrane was washed with TBS-T and then incubated with anti-mouse and anti-rabbit secondary antibodies conjugated to horseradish peroxidase for 1 h at room temperature. The detection of antibody binding was performed with Pierce ECL Western Blotting Substrate (Thermo Scientific, Waltham, MA, USA) and images were acquired with an Alliance Mini HD9 (Uvitec, Cambridge, UK). Densitometric analysis was performed with ImageJ software (https://imagej.nih.gov/ij/download.html).

#### 2.8.7. Fluorescence Microscopy

Cells cultured on the different Ti coatings were fixed with 4% paraformaldehyde in 0.1 M phosphate-buffered saline (PBS), pH 7.4, at 4 °C for 30 min. After washing twice with PBS, cells were permeabilized with 0.1% Triton X-100 in 0.01 M PBS to remove the nuclear envelope and soluble nuclear material and were then blocked with normal goat serum in PBS (dilution 1:5). Cells were incubated for 45 min at room temperature with TRITC-labeled phalloidin (dil 1:100) to visualize F-actin fiber organization (red fluorescence) and with DAPI (dil 1:1000) to stain cell nuclei (blue fluorescence). All samples were immersed in mounting medium (VECTASHIELD^®^), before laying on a coverslip and visualizing them under a fluorescent microscope (Nikon Eclipse 600, Milan, Italy) equipped with NIS-Elements microscope imaging software (Nikon). For the analysis of the stress fiber formation, a five-point scoring system measuring the degree of actin stress fiber was used [16]. The criteria for blind scoring were: (1) little or no resolved F-actin stress fiber formation and mostly cortical actin; (2) thin, short F-actin filaments generally occupying at least 25% of the cell volume; (3) moderate stress fiber formation of F-actin where stress fibers are thicker and occupy at least 50% of the cell volume; (4) extensive stress fiber formation where stress fibers are thick and well defined; many stress fibers traversing the full width of the cell; (5) the entire cell is densely packed with thick stress fibers, most traverse the width of the cell. At least 20 cells were counted for each substrate. Mean data ± SD are reported.

### 2.9. Statistical Analyses

Physico-chemical characterizations were performed in triplicate, expressing results as mean ± standard deviation. Biological experiments were carried out using six sample replicates. The statistical analysis of data was performed with GraphPad Prism software (v.8.4.1). Data were compared by ANOVA, followed by Tukey’s test. As far as biological experiments were concerned, the measurement errors were taken from three replicates of two different experiments. Data were analyzed by Mann–Whitney U tests. For all data, statistical significance was declared at *p* < 0.05.

## 3. Results and Discussion

### 3.1. Arbutin Cytocompatibility and Antioxidant Activity

The tmplant environment could cause oxidative stress and the modulation of antioxidants by surface modification could improve the osseointegration of Ti-based implants. In this study, biological tests were first devoted to assessing the effect of different arbutin concentrations on osteoblast behavior, to define the appropriate amount of arbutin to be loaded in a polymeric coating on titanium endowed with antioxidant properties. Among the different osteoblast cell lines used for the implant biocompatibility assessment, Saos-2 cells are widely employed based on their cell anchorage dependency and homogeneity [26]. Moreover, to closely simulate implantation conditions, human PDPCs were also tested.

**Figure 1:**
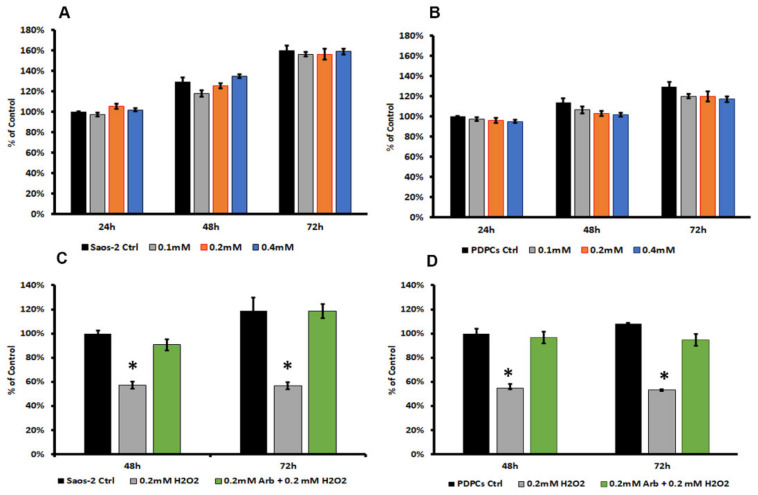
3-dimethylthiazol-2,5-diphenyltetrazolium bromide (MTT) viability test: histograms of Saos-2 (**A**) and periosteal derived precursor cells (PDPCs) (**B**) cultured with different concentrations of arbutin for up to 72 h. Histograms of Saos-2 (**C**) and PDPCs (**D**) cultured with 0.2 mM of arbutin and exposed to oxidative stress (48 h = 24 h after stress induction, 72 h = 48 h after stress induction). Data are expressed as the percentage of Saos-2 or PDPCs cultured without arbutin after 24 h (control); * *p* < 0.05 vs. ctrl.

The MTT assay showed that the tested arbutin concentrations did not hamper the viability of both cell populations during up to 72 h of contact (Figure 1A,B). No significant differences in terms of concentration effect were detected. Thus, considering in vitro antioxidant activity (i.e., DPPH assay, Section 3.3), we decided to test the capability of 0.2 mM arbutin, loaded into a polymeric coating, to restore cell viability after stress induction. To the latter purpose, we added increasing concentrations of H_2_O_2_ (from 0.025 mM to 0.3 mM) in culture media to define the appropriate H_2_O_2_ amount to use on both cytotypes (See Supplementary Materials Figure S1). The H_2_O_2_ concentration was set at 0.2 mM, as it was capable of inducing a reduction in cell viability of about 40–60% in both cell types. The previous addition of 0.2 mM arbutin to the culture media was able to restore cell viability in SaoS-2 (Figure 1C) and PDPCs (Figure 1D) after stress induction at both the timepoints analyzed.

As far as PDPCs are concerned, further investigations were performed to ascertain if arbutin could affect gene and protein expression, evaluating the main genes involved in osteoblastic commitment. To shed light on this aspect, the experiments were made both in normal and osteogenic conditions. The mesenchymal stem cell fate toward the osteoblast lineage is normally achieved by inducing the osteogenic transcription factors RUNX-2 by BMP2. These immature cells still have the potential to divide and express low levels of alkaline phosphatase (ALP) activity and to synthesize a low amount of type I collagen. The further differentiation of these cells is dependent on a sequential increased expression of ALP and several non-collagenous proteins, such as osteonectin, osteopontin and osteocalcin, which have fundamental effects on the newly laid bone matrix maturation and mineralization [27].

The qRT-PCR relative expression analysis suggested the capability of arbutin to modulate the expression of genes involved in the early (i.e., bmp2, runx-2 and alp) and late (i.e., collagen and sparc) differentiation towards an osteoblastic phenotype at both time points analyzed. Relative expression data are summarized in Supplementary Materials Table S2.

**Figure 2:**
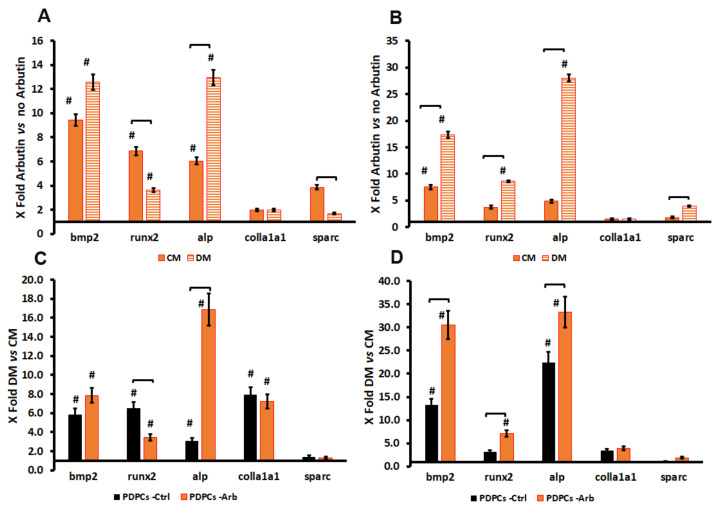
Histograms depicting changes in bmp2, runx-2, alp, collagen type I and sparc mRNA in PDPCs cultured with or without arbutin in complete (CM) or differentiating (DM) medium for 7 (**A**,**C**) and 14 days (**B**,**D**). (**A**,**B**) Data are expressed as fold change (2^−ΔΔCt^) as compared to untreated cells (i.e., arbutin vs. no arbutin), The axes intersect at 1, which indicates the mRNA expression in untreated cells. (**C**,**D**) Data are expressed as fold change (2^−ΔΔCt^) as compared to cells cultured in CM (i.e., DM vs. CM), the axes intersect at 1, which indicates the mRNA expression in cells cultured in CM. # indicates significant (*p* < 0.05) differences in comparison to controls and square brackets indicate significant differences between the analyzed groups.

To better point out the possible interference of arbutin on PDPC differentiation potential, mRNA changes were also examined using the ΔΔCt method [25], comparing values obtained in cells cultured with arbutin with those of untreated ones (Figure 2). After 7 days, the presence of arbutin induced a significant upregulation of all genes of early osteoblastic differentiation in comparison with untreated cells. A significant upregulation of the mRNA expression for alkaline phosphates (alp), as well as lower values of runx-2 and sparc, were detected in differentiating medium (DM)-treated cells in comparison to those cultured in complete medium (CM). These data are consistent with the use of a medium (i.e., DM) made of dexamethasone, ascorbic acid and β-glycerophosphate, whose combination increased alp levels in in vitro cultures [28]. The addition of an exogenous factor (i.e., arbutin) seems to focus cell bioenergetics on ALP synthesis, limiting the expression of other markers. No changes were evident in the expression of collagen type I mRNA (Figure 2A).

After 14 days of culture, the upregulation of genes was higher than after 7 days, except for collagen type I mRNA expression. Moreover, an upregulation of the mRNA for sparc was detected in cells cultured in DM (Figure 2B), suggesting an increase in the level of differentiation and the effective cell adaptation to the proposed culture conditions. Overall, the qRT-PCR results indicated that arbutin is able to modulate the gene expression induced by an osteogenic medium in PDPCs, supporting their differentiation towards an osteoblastic phenotype. These results were highlighted when expressed (ΔΔCt method) in cells cultured in DM were compared with those in CM (Figure 2C,D).

**Figure 3:**
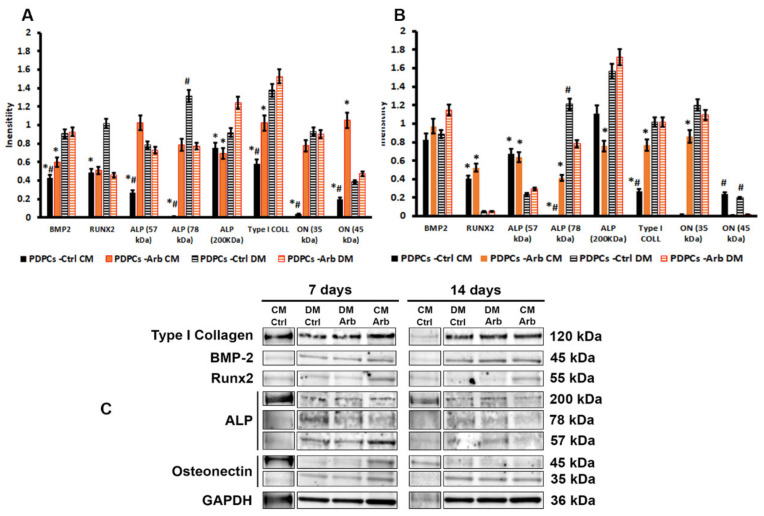
Histograms depicting densitometric quantitation of western blotting at 7 (**A**) and 14 (**B**) days of culture of the gel presented in (**C**): results are expressed as intensity normalized to GAPDH. Asterisks indicate significant differences between CM and DM (*p* < 0.05); # indicates significant differences between ctrl and arbutin (*p* < 0.05).

Based on these data, we analyzed the expression of the proteins whose genes were upregulated by arbutin. Western blotting analyses, depicted in Figure 3, highlighted the role of arbutin in all culture conditions analyzed. In osteogenic conditions (DM) a significant (*p* < 0.05) reduction in RUNX-2 and ALP (78 kDa fragment) protein content and an increase in ALP 200 kDa expression were seen after 7 days of culture with arbutin, in comparison with untreated cells (Figure 3A). After 14 days, an increase in proteins involved in the early osteoblastic differentiation was detected, concomitantly with a reduction in the amount of ON (Figure 3B).

The human ALP peptide is synthesized as a native protein with a molecular weight of 57 kDa and it is then modified in the endoplasmic reticulum and in the Golgi apparatus, with the addition of sugar chains, until it reaches the mature form of about 80 kDa. The functional ALP enzyme is assumed to exist as a homodimer with a molecular weight of about 165–200 kDa [29,30,31]. Similarly, osteonectin is detectable as two different fragments, where the 45 kDa fragment represents the active form [32]. After 14 days of culture, our densitometric analysis showed a substantial reduction in the functional form of ON, but not of the immature one, and no significative variations in the expression of the ALP enzyme. Our data are consistent with the idea that arbutin did not hamper the capability of PDPC differentiation towards the osteoblastic phenotypes. Arbutin may favor the anabolic activity of human precursor cells and also support their differentiation [9].

### 3.2. Coating Preparation, Morphological and Physicochemical Characterization

#### 3.2.1. Electrochemical Preparation of PEGDA-AA/Arb Coatings on Titanium

To obtain PEGDA-AA coatings on Ti electrodes, an electro-reductive process was applied using the cyclic voltammetry technique. In particular, the potential was cycled between 0.0 V and −1.2 V for 20 cycles (scan rate = 100 mV/s). Arbutin was loaded during or after electrosynthesis (PEGDA-AA DE and PEGDA-AA AE, respectively), for further details, see Section 2.3. The addition of arbutin during the PEGDA-AA electropolymerization did not compromise in any way the film growth, as shown by the cyclic voltammograms with and without arbutin (see Supplementary Materials Figure S3). Some of the prepared coatings, with or without arbutin loading, were subjected to an annealing treatment to promote the polymeric network rearrangement under a thermal trigger. All coatings, annealed and unannealed, were deeply characterized to shed light on the suitability of the thermal treatment.

#### 3.2.2. Scanning Electron Microscopy of the Coatings

**Figure 4:**
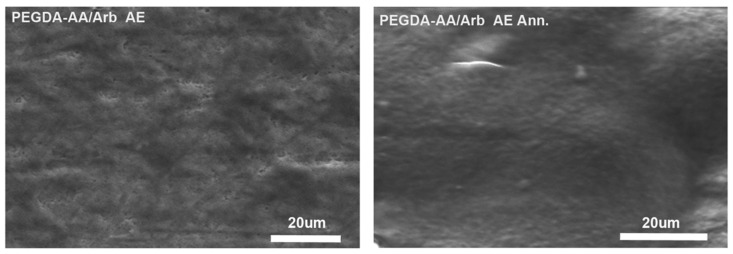
SEM morphological analyses of PEGCA-AA/Arb AE, as prepared (**on the left**) and after annealing (**on the right**).

PEGDA-AA/Arb AE coatings, as prepared or after annealing, underwent morphological characterization through SEM. Micrographs in Figure 4 display the impact of the annealing procedure on the coating topography. First, the PEGDA-AA/Arb AE coatings, as prepared, had irregular morphology, reflecting the grooves and scratches of the underlying titanium. Conversely, a noteworthy enhancement of the film homogeneity can be seen after the annealing treatment.

#### 3.2.3. XPS Analysis of the Coatings

PEGDA-AA-based coatings were characterized using the XPS technique, to ascertain the surface composition and the arbutin presence on the coatings. The elemental atomic percentages of the analyzed samples were reported in Table 1.

The C/O corrected peak area ratios, relevant to arbutin and PEGDA-AA samples (i.e., 1.7 for arbutin, 2.1 and 1.9 for unannealed and annealed copolymer coatings, respectively), were equal in both cases to the theoretical ones (i.e., 1.7 for arbutin and 2.0 for the copolymer).

**Figure 5:**
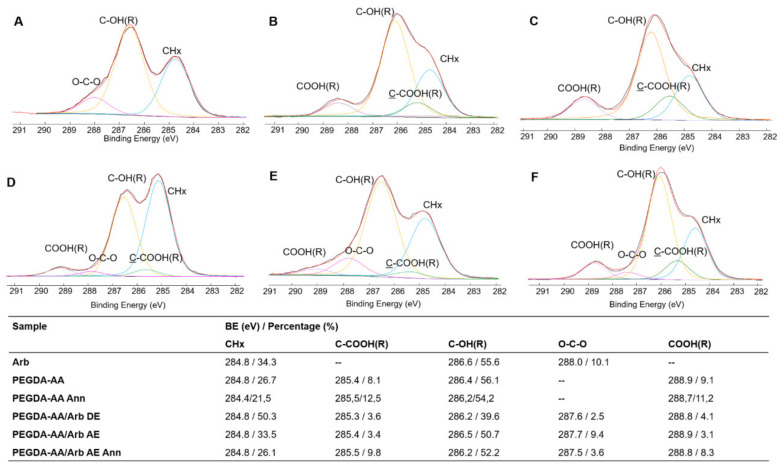
Curve fitting of C1s signals of (**A**) pure arbutin and (**B**) PEGDA-AA, (**C**) PEGDA-AA Ann., (**D**) PEGDA-AA/Arb DE, (**E**) PEGDA-AA/Arb AE and PEGDA-AA/Arb AE Ann. (**F**) Coatings electrosynthesized on Ti sheets. Attributions, binding energies values and percentages are reported in the table.

As far as the C1s high-resolution spectra are concerned, detailed curve fittings of the analyzed samples are reported in Figure 4, together with the relevant attributions and binding energies. In the pure arbutin C1s curve fitting (Figure 5A), the first peak was attributed to the C–H, C-C aromatic bonds with a characteristic binding energy (BE) of 284.8 eV, in addition to the ubiquitous hydrocarbon contamination. The second peak was ascribed to the C–O bond in the hydroxyl or ether groups at 286.6 eV. The peak at a BE of 288.0 eV was attributed to the anomeric carbon linked to two oxygen atoms, i.e., an O-C-O bond, typical of glucosidic molecules.

To study the composition of the PEGDA-AA copolymer (Figure 5B), the peak area ratio between C-OH(R):COOH(R) was compared to that observed in the used macromer (i.e., PEGDA_575_) equal to 7.4:1 [33]. The PEGDA-AA system showed a lower ratio (6.2:1), justified by the presence of the AA that increased the COOH(R) peak. For PEGDA-AA annealed (Figure 5C), this ratio was further decreased to 4.8:1, suggesting a surfacing of COOH groups due to the thermal reorganization, mainly involving the mobility of AA moieties [17].

Moreover, PEGDA-AA/Arb DE, AE and AE Ann. coatings (Figure 5D–F) showed the additional O-C-O contribution, suggesting the presence of arbutin on the surface of the samples. Considering that this functionality was present only in the glucosidic compound, and the carboxylic groups belong only to the copolymer, a comparison of the O-C-O:COOH(R) peak area ratios can supply an estimation of the arbutin surface allocation in the three coatings. In particular, in PEGDA-AA/Arb DE, a ratio of 0.6:1 was calculated. When arbutin was loaded after electrosynthesis, a high surface allocation was recorded, with O-C-O:COOH(R) equal to 3:1. Finally, when the latter sample was subjected to annealing, this ratio lowered to 0.4:1, thus evidencing a low arbutin surface allocation in the annealed specimen, due to the chain reorganization after the heat treatment. On the other hand, the HPLC results (see Section 3.2.4) highlighted a similar arbutin content in the PEGDA-AA/Arb AE and PEGDA-AA/Arb AE Ann. coatings, allowing us to conclude that most of the phytochemical was present in deeper layers of the annealed system. Furthermore, to assess the impact of the annealing procedure on arbutin stability, TGA analyses were performed (Supplementary Materials Section 2.4). The thermogram of arbutin evidenced a substantial stability of the molecule up to 238 °C (T_onset_ of the first decomposition step of arbutin), thus suggesting that no thermal decomposition occurred at the annealing temperature (i.e., 200 °C).

#### 3.2.4. Arbutin Quantification by High-Performance Liquid Chromatography (HPLC)

The preliminary biological assessments revealed that a 0.2 mM concentration of the phytochemical was useful to ensure a protective effect against H_2_O_2_ without altering cell behavior (see Section 3.1). Therefore, several strategies were set up to load the proper amount of arbutin onto the prepared coatings. First, arbutin was added during the electrosynthesis of the coating (PEGDA-AA/Arb DE), to allow a homogeneous incorporation of the phytochemical into the growing polymer. However, HPLC analyses revealed that only a tenth of the desired arbutin amount was loaded on PEGDA-AA/Arb DE coatings (Table 2). Hence, this approach was dismissed. As a promising alternative, arbutin was added after the film electrosynthesis (PEGDA-AA/Arb AE), dipping it into a solution of the phytochemical compound, as described in Section 2.3. The most promising results were obtained with a 0.01 M arbutin solution, which allowed the loading of up to 0.41 mM of arbutin into the polymeric systems. Therefore, setting the incubation time of the coated titanium substrate in the 0.01 M arbutin solution to 1 h, the desired arbutin loading of about 0.02 mM was achieved (Table 2). Furthermore, HPLC analyses demonstrated that the annealing treatment did not change arbutin loading on the polymeric coatings (refer to PEGDA-AA/Arb AE 1 h and PEGDA-AA/Arb AE 1 h Ann. coatings in Table 2). Nevertheless, the XPS results suggested that the annealing treatment promoted arbutin distribution in the coating’s depth, likely due to a rearrangement of the system’s polymeric chains (see Section 3.2.3).

This restructuring of the polymeric network could also be responsible for the slightly slower arbutin release, observed from PEGDA-AA/Arb AE Ann. with respect to PEGDA-AA/Arb AE. In both cases, however, the phytochemical compound was completely released within 8 h. Additional details on arbutin’s in vitro release from the coatings in physiological conditions are reported in the Supplementary Materials Section 2.5.

### 3.3. Antioxidant Activity Evaluations by DPPH Assay

**Figure 6:**
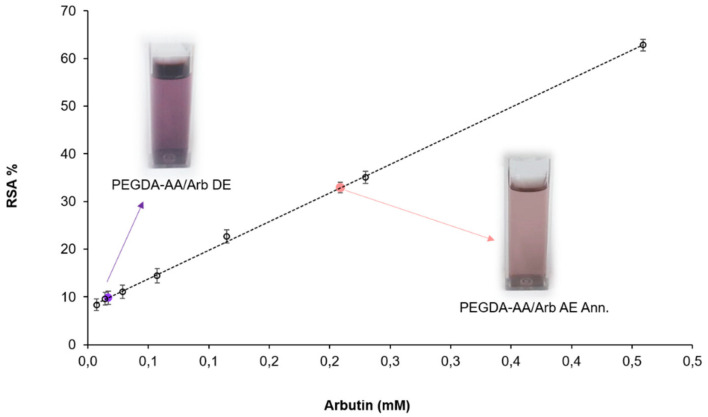
DPPH assay to assess in vitro antioxidant activity. The calibration curve (r^2^ 0.999) was obtained with arbutin-containing solutions at decreasing concentrations (from 0.5 mM to 8 µM). The cuvettes refer to PEGDA-AA/Arb DE and PEGDA-AA/Arb AE Ann.

The DPPH assay displayed the in vitro antioxidant activity of the arbutin-loaded coatings. After incorporation in PEGDA-AA, the phytochemical compound retained its scavenger features, eliciting a concentration-dependent antioxidant effect (Figure 6). PEGDA-AA/Arb DE was able to load only 0.02 mM of arbutin, thus reaching 9.8 ± 0.3%RSA. On the other hand, the highest radical scavenging activity (33.50 ± 0.04%RSA) was achieved with PEGDA-AA/Arb AE Ann., which trapped 0.2 mM of arbutin. Furthermore, the PEGDA-AA/Arb AE as prepared, without annealing, reached an antioxidant activity of 32.9 ± 0.1%RSA. Therefore, it could be concluded that the annealing procedure did not alter the arbutin antioxidant performance. Furthermore, the %RSA of PEGDA-AA/Arb AE Ann. coating overcame that of other antioxidant systems reported in the literature. Indeed, Catauro et al. reported a 24.7%RSA against DPPH for silica/poly(ε-caprolactone) implants trapping 15%wt quercetin. The authors found, at 24.7%RSA, the suitable balance between cytotoxicity and oxidative stress prevention [34]. Furthermore, Shrikanta et al. explored the antioxidant activity of 0.2 mg/mL of mulberry fruit extracts with the DPPH assay, measuring an activity of 20.08% of DPPH radicals, which increased to 77.75% at 1.0 mg/mL [20].

Considering the scavenging performances of PEGDA-AA/Arb AE Ann. coatings, together with their improved morphology due to annealing, as well as arbutin loading and release behavior, the PEGDA-AA/Arb AE Ann. coatings proved to be the best systems prepared. Therefore, they were selected for the subsequent biological experiments, described below.

### 3.4. Arbutin-Loaded Coating Cytocompatibility and Anti-Oxidant Activity

To closely simulate implantation, tests on PEGDA-AA/Arb AE Ann. were performed with human PDPCs. At first, investigations evaluated the cell viability and adhesion using MTT tests at 48 and 72 h after seeding (24 h and 48 h after oxidative stress induction, respectively) and cytoskeletal detections at 48 h (24 h after oxidative stress induction). Then, the effect of the PEGDA-AA/Arb AE Ann. on PDPC early differentiation towards an osteoblastic phenotype was assessed by culturing cells for up to 7 days. All investigations were also performed in the presence of oxidative stress.

**Figure 7:**
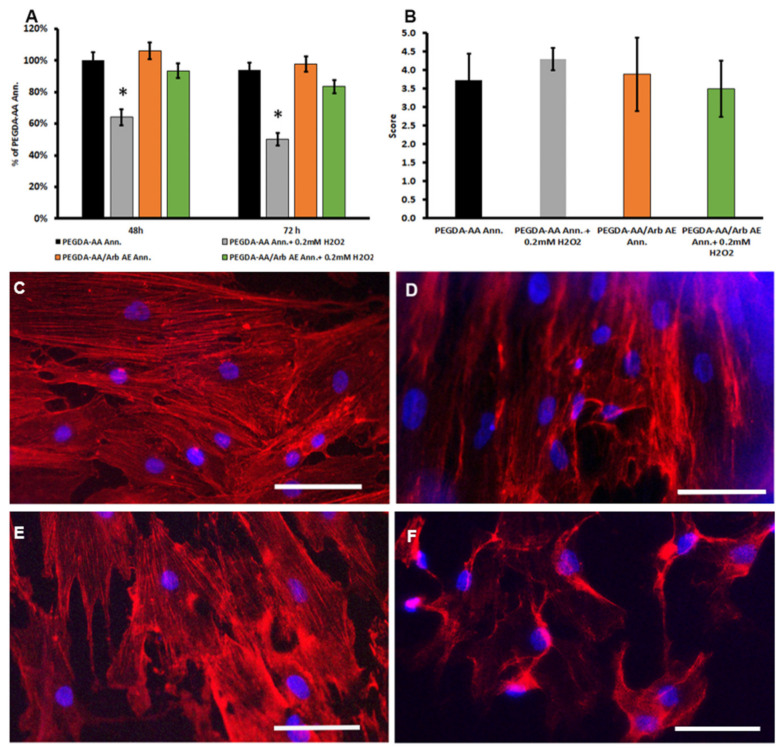
(**A**) MTT viability test in PDPCs cultured on PEGDA-AA Ann. and PEGDA-AA/Arb AE Ann. and exposed to oxidative stress. Data are expressed as a percentage of PDPCs cultured on PEGDA-AA Ann. 48 h = 24 h after stress induction, 72 h = 48 h after stress induction, * *p* < 0.05 vs. PEGDA-AA Ann. (**B**) Histogram depicts the semiquantitative analysis of the actin cytoskeleton. (**C**–**F**) Representative images of actin cytoskeleton immunofluorescence detection in PDPCs cultured on PEGDA-AA Ann. without (**C**) or with (**D**) oxidative stress, and PEGDA-AA/Arb AE Ann. without (**E**) or with (**F**) oxidative stress. Scale bars = 50 μm.

The MTT tests evidenced the good viability of PDPCs cultured on the coatings with arbutin, with the maintenance of values higher than 80% after the administration of 0.2 mM H_2_O_2_ in the culture medium. On the contrary, on PEGDA-AA Ann., the addition of H_2_O_2_ significantly reduced the percentage of viable cells (Figure 7A).

The modification of cell shape gives important clues concerning the role of surface chemistry and morphology on cell−substrate interactions. For this reason, it is worth considering what the shapes of osteoblast-like cells look like when they are grown on differently coated substrates. To react to different surfaces, cells sense shape through different cell structures (stress fiber formation, lamellipodia and filopodia), which are responsible for initializing and transmitting the effect of the surface throughout the cell, influencing cell functions. Filopodia, cytoplasmic protrusions that are extended by cells at their leading edges, are involved in gathering special information, are crucial in cell migration and serve as topographical sensors to detect the immediate surrounding environment [35].

Cytoskeletal fluorescence detection, depicted in Figure 7, compares how cells adhere to the titanium coatings with (PEGDA-AA/Arb AE Ann.) or without (PEGDA-AA Ann.) arbutin after 48 h. The images clearly show the presence of actin stress fibers and focal adhesions in the spread PDPCs on the analyzed coatings. As far as semiquantitative analysis is concerned (Figure 7B), PDPCs cultured both on PEGDA-AA Ann. and PEGDA-AA/Arb AE Ann. displayed a high spreading, with spindle-shaped cells containing well-defined cytoskeletal organization and many stress fibers (scores 3.7 ± 0.7 and 3.9 ± 0.8, respectively). Due to the high dimension of cells, cell–cell contacts were also present. Overall, this reflects a strong influence of primary adhesion conditions on the future cell fate, which seems to be unaffected by the presence of arbutin. The addition of H_2_O_2_ for 24 h in cell cultured on PEGDA-AA Ann. evidenced the presence of cells larger in dimension and an increase in stress fiber formation (score 4.3 ± 0.3), features suggesting cell suffering. On the contrary, cells cultured on PEGDA-AA/Arb AE Ann. showed no significant modifications in cell dimension and cytoskeletal organization (score 3.5 ± 0.7) in comparison with unstressed cells, suggesting a beneficial effect of the presence of arbutin, even if a slight reduction in cell number was detected.

**Figure 8:**
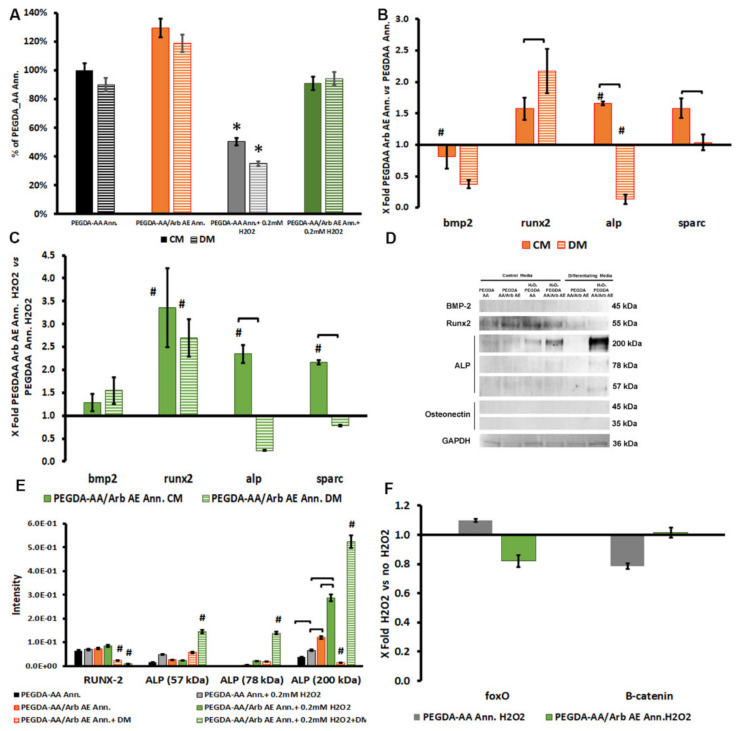
(**A**) MTT viability of PDPCs cultured on PEGDA-AA Ann. and PEGDA-AA/Arb AE Ann. and exposed to oxidative stress for up to 7 days of culture. Data are expressed as a percentage of PEGDA-AA Ann.; * *p* < 0.05 vs. all conditions. (**B**) Histogram depicting changes in bmp2, runx-2, alp and sparc mRNAs in PDPCs on PEGDA-AA/Arb AE Ann. cultured in complete (CM) or differentiating (DM) medium for 7 days. Data are expressed as fold change (2^−ΔΔCt^) over PEGDA-AA Ann. The axes intersect at 1, which indicates the range of mRNA expression in cells cultured on PEGDA-AA Ann. (**C**) Histogram depicting changes in bmp2, runx-2, alp and sparc mRNAs in PDPCs on PEGDA-AA/Arb AE Ann. cultured in complete (CM) or differentiating (DM) medium both in the presence of H_2_O_2_ for 7 days. Data are expressed as fold change (2^−ΔΔCt^) over PEGDA-AA Ann. in the presence of H_2_O_2_. The axes intersect at 1, which indicates the range of mRNA expression in untreated cells; # indicates significant differences (*p* < 0.05) in comparison to controls without arbutin and square brackets indicate significant differences between cells cultured in CM and DM. (**D**) Western blot analysis protein expression. (**E**) Histograms depicting the densitometric quantitation of RUNX-2 and ALP of the blots presented in D. Since BMP-2 and ON were not expressed, they were not considered for the histogram. (**F**) FoxO and β-catenin variations in PEGDA-AA Ann. and PEGDA-AA/Arb AE Ann. in cells undergoing stress induction. Data are expressed as fold change (2^−ΔΔCt^) of H_2_O_2_ untreated cells; the axes intersect at 1, which indicates the range of mRNA expression in untreated cells.

MTT viability tests on the different titanium coatings after 7 days confirmed that the presence of arbutin on the coatings was capable of hampering the oxidative stress induced by the presence of H_2_O_2_, enhancing cell viability. This effect was also maintained in osteogenic conditions (Figure 8A). As for the effect of the PEGDA-AA/Arb AE Ann. on PDPC early differentiation towards an osteoblastic phenotype, the presence of arbutin seemed to support the expression of some osteoblastic differentiation markers, as suggested by the upregulation of runx-2, and the downregulation of alp mRNAs concomitant with the production of the ALP protein fragments (Figure 8). In agreement with the previous experiment, the qRT-PCR mRNA changes were examined using the ΔΔCt method [25] to better point out the possible interference of arbutin (Figure 8B) or H_2_O_2_ (Figure 8C) on PDPC differentiation potential. Relative expression data are summarized in Supplementary Materials Table S3. Interestingly, the ALP protein expression seemed to be enhanced by the presence of H_2_O_2_ (Figure 8D,E). ALP is one of the early osteoblastic markers that occurs in mature osteoblast progenitors and is upregulated until the differentiation is well progressed and the proliferation ceases [36,37].

Arbutin is a chain-breaking antioxidant able to eliminate reactive radicals whose protective role against H_2_O_2_-induced injury in vitro was already investigated [8,38]. Following the abovementioned results, we can hypothesize that arbutin can both combat the H_2_O_2_ oxidant effect and stimulate osteoblastic differentiation, promoting the expression of osteoblastic markers, such as ALP. In line with the behavior of ALP, the differences observed in the mRNA expression of the other genes involved in osteoblastogenesis in cells cultured under different stimulating conditions (i.e., CM vs. DM) are suggestive of a speeding up of differentiation in the presence of arbutin (Figure 8B). Comparing these observations with the data obtained with arbutin alone after 7 days, we observed differences concerning bmp2 mRNA and protein expression, which was downregulated and undetectable in cells cultured on the PEGDA-AA/Arb AE Ann. coating. As stated above, osteoblastic differentiation is a sequentially triggered phenomenon that starts from bmp2. It can be speculated that the presence of polyacrylates may play a role in modulating the differentiation of PDPCs, providing a further stimulus towards osteoblastic commitment [39].

To get insights into the possible modulation of PDPC behavior under oxidative stress, the mRNA expression of FoxO and β-catenin was also analyzed.

FoxO promotes cell survival, by cell cycle arrest in the G1 phase, inducing quiescence and regulating longevity in model organisms. FoxO-mediated transcription requires the binding of β-catenin, a scaffold protein that is also involved in osteoblastogenesis by the Wnt/β-catenin/TCF pathway [40]. The presence of ROS could antagonize the differentiation process, allowing the formation of a FoxO/βcatenin complex, thus reducing the capability of TCF-β-catenin binding, an essential step for the β-catenin nuclear translocation and the consequent runx-2 transcription [41].

In MSCs, a basal oxidative level is required to enhance osteogenesis and calcification, as low levels of ROS act as second messengers on several different molecular pathways [42]. ROS and oxidative stress may, however, decrease the procedure of osteogenic differentiation, and the addition of H_2_O_2_ reduces the in vitro osteogenic differentiation of MSCs and OB precursors (i.e., PDPCs) [43].

We observed that, after oxidative stress induction, mRNA for FoxO was downregulated in cells cultured on PEGDA AA/Arb AE Ann. in comparison to cells on PEGDA-AA Ann. (Figure 8F), whilst β-catenin was slightly downregulated only in PEGDA-AA Ann. FoxO is generally overexpressed when cells must respond to the stress to safeguard their survival. Therefore, its downregulation in PDPCs cultured on PEGDA AA/Arb AE Ann. supported the hypothesis of arbutin’s scavenger activity [38], which created an optimal environment for the cells, providing only the basal oxidative level required for differentiation. We can also speculate that arbutin embedded in the coating, in the presence of H_2_O_2_, in differentiating conditions, can hamper the stress, maintaining the β-catenin signaling towards the osteoblastic differentiation of PDPCs [44], thus also promoting implant osseointegration in an inflamed microenvironment. Data on the osteogenic commitment of PDPCs on PEGDA AA/Arb AE Ann. under oxidative stress conditions (Figure 8C) supported this consideration, showing the same trend of cells in a non-oxidizing environment (Figure 8B). This assumption is also related to the fact that one of the main targets in the oxidative stress-induced inhibition of MSCs and osteoblast differentiation is RUNX-2 phosphorylation [45,46]. The presence of arbutin still allowed the production of the latter molecule and, consequently, PDPC osteoblastic differentiation.

## 4. Conclusions

In this work, for the first time, the role of arbutin on the viability of Saos-2 and PDPCs was demonstrated, highlighting its involvement in hampering oxidative stress and restoring cell viability, while maintaining a correct cell morphology. Furthermore, we proved that arbutin modulates in vitro gene and protein expression in PDPCs, playing a protective effect during their osteoblastic differentiation. As far as arbutin-loaded PEGDA-AA Ann. coatings on titanium implants are concerned, they were deeply characterized in terms of surface composition, loading and the release of the active molecule. The biological results showed a good interaction of osteoblast precursor cells with the proposed coatings and suggested high biocompatibility. Besides, the released arbutin supported the early osteoblastic commitment of PDPCs, also under oxidative stress conditions, opening intriguing opportunities for the development of antioxidant coatings on titanium implants.

## Figures and Tables

**Table 1:** Atomic composition of the samples by means of XPS analysis.

Sample	Atomic Percentages (%)	
	C1s	O1s
Arb	63.4	36.6
PEGDA-AA	67.6	32.4
PEGDA-AA Ann.	66.1	33.9
PEGDA-AA/Arb DE	72.8	23.6
PEGDA-AA/Arb AE	65.8	34.2
PEGDA-AA/Arb AE Ann.	69.9	30.1

**Table 2:** Arbutin loading onto the prepared coatings, evaluated by HPLC.

Sample	Loaded Arbutin (mM)
PEGDA-AA/Arb DE	0.019 ± 0.002
PEGDA-AA/Arb AE 1 h	0.220 ± 0.004
PEGDA-AA/Arb AE 1 h Ann.	0.206 ± 0.003
PEGDA-AA/Arb AE 3 h	0.41 ± 0.09

## Supplementary Materials

The Supplementary Materials file is available online at https://www.mdpi.com/2076-3921/9/7/579/s1.

## Abbreviations

AA	Acrylic acid
ALP	Alkaline phosphatase
BMP2	Bone morphogenetic protein 2
Coll1	Collagen type I
CM	Complete medium, i.e., DMEM with 10% FBS
DM	Differentiating medium, i.e., DMEM supplemented with the osteogenic factors: dexamethasone, ascorbic acid and β-glycerophosphate
DMEM	Dulbecco’s modified Eagle’s medium
DPPH assay	2,2-difenil-1-picrylhydrazyl assay
FBS	Fetal bovine serum
FoxO	Forkhead box O
GAPDH	Glyceraldehyde-3-phosphate dehydrogenase
GUSB	Beta-glucuronidase
HPLC	High-performance liquid chromatography
MSCs	Mesenchymal stem cells
MTT	3-dimethylthiazol-2,5-diphenyltetrazolium bromide
OB	Osteoblasts
ON	Osteonectin–protein
PDPCs	Periosteal-derived precursor cells
PEGDA	Poly(ethylene-glycol diacrylate)
PEGDA-AA	Poly(acrylic acid)–poly(ethylene-glycol diacrylate) coating
PEGDA-AA/Arb AE	Poly(acrylic acid)–poly(ethylene-glycol diacrylate) coating with arbutin embedded after electrosynthesis
PEGDA-AA/Arb DE	Poly(acrylic acid)–poly(ethylene-glycol diacrylate) coating with arbutin embedded during electrosynthesis
RANK-L	Receptor activator of nuclear factor kappa-Β ligand
RSA %	Radical scavenging activity percentage
ROS	Reactive oxygen species
RUNX-2	Runt-related transcription factor 2
Saos-2	Human osteosarcoma cell line
SEM	Scanning electron microscopy
Sparc	Secreted protein, acidic, cysteine-rich–osteonectin gene
TCF	T-cell factor
TGA	Thermogravimetric analysis
XPS	X-ray photoelectron spectroscopy
Wnt	Wingless-related integration site
